# Utility of the Medical Knowledge AI Copilot, OpenEvidence: A Patient With 100 Cerebral Microhemorrhages

**DOI:** 10.7759/cureus.108098

**Published:** 2026-05-01

**Authors:** Amber N Navarra, Salman Ikramuddin, Steven M Greenberg, P. Murali Doraiswamy

**Affiliations:** 1 Psychiatry, Duke University School of Medicine, Durham, USA; 2 Neurology, Duke University School of Medicine, Durham, USA; 3 Neurology, Harvard Medical School, Boston, USA

**Keywords:** alzheimer’s dementia, artificial intelligence in medicine, cerebral amyloid angiopathy (caa), large language models (llm), mild cognitive impairment (mci), openevidence

## Abstract

Cerebral amyloid angiopathy (CAA) is a highly prevalent condition that often poses diagnostic and therapeutic challenges, especially in people with comorbid mild cognitive impairment (MCI). OpenEvidence (OE; Miami, Florida, US), a leading free medical knowledge copilot, reports it has supported over 100 million Artificial Intelligence (AI)-powered clinical consultations from U.S. doctors and other frontline clinicians. The objective of this study is to use a single case with two common comorbid neurological conditions to retrospectively appraise the ability of OE to serve as a clinically relevant knowledge aid. We input the de-identified summary of a case of probable CAA and MCI in OE. Overall, the AI recommendations very closely matched the diagnosis and care that was actually provided. We identified some areas (e.g., prognostication, geriatric risk-benefit trade-offs, diagnostic terminology) where it could be improved to match the logic and knowledge of a subspecialist. OE used as a knowledge copilot can help inform point-of-care physician decisions by giving them more information than they may have otherwise considered.

## Introduction

Artificial Intelligence (AI) tools based on large language models (LLMs) are rapidly impacting healthcare through a combination of high speed and information synthesized from the internet’s vast sources [1-6]. OpenEvidence (OE; Miami, Florida, United States) has quickly become one of the most widely used LLM medical knowledge copilots used at the point of care, with over one million queries daily [1-4]. It is available for free for qualified medical professionals and offers information curated from highly trusted sources such as top medical journals [2-4]. OE has a user-friendly interface (similar to Google Search), and users typically find its answers to be quick, fast, clear, highly relevant, and well-supported by multiple high-quality references and graphs. A prior study of five cases at the Mayo Clinic found OE to perform comparably to physicians and provide accurate, evidence-based responses that align with those of physicians [3]. It was shown to be a tool that would aid physicians with decision-making and help bridge knowledge gaps [3]. OE reports that to date it has supported over 100 million AI-powered clinical consultations from U.S. doctors and other frontline clinicians [4].

AI LLMs can also be vulnerable to multifaceted risks in patient care [5,6]. These include hallucinations, misinformation, biases, or incomplete information leading to suboptimal or wrong patient care, which in turn could lead to adverse outcomes as well as privacy risks [5,6]. Trainees, who tend to rely more on AI LLMs, may be particularly vulnerable to these risks. While OE has licensed and curated data from some of the most trusted medical sources [3-5], the practice of medicine has many nuances and may require logic and reasoning that require specialized expertise. Hence, it is vital to continuously monitor the outputs of AI in multiple different diseases and settings [5,6].

The objective of our study was to retrospectively evaluate the utility of OE as a knowledge co-pilot to make diagnostic, prognostic, and treatment recommendations for a patient with two comorbid neurological conditions and to compare it with decisions actually made by the specialists who treated the case.

## Materials and methods

OE (version 2.8.24) was accessed via its online portal on March 26, 2026. To evaluate the utility of OE, we used a case of an elderly man with cerebral amyloid angiopathy (CAA) and mild cognitive impairment (MCI) whose diagnostic and treatment details were published [7]. This patient was selected due to the comorbidity of two common neurological conditions. This patient was treated clinically by specialists without the use of AI. We then retrospectively input a summary of his case to evaluate the performance of OE across five broad concordance domains: diagnostic evaluation, diagnosis, differential diagnosis, treatment plan, and prognosis. The platform’s responses were then compared for concordance with what was actually clinically done by a specialist. We evaluated whether the diagnoses provided by OE matched the exact diagnosis (probable CAA and MCI due to Alzheimer's disease) given by the treating physician. We also evaluated whether the treatments proposed by OE matched the way the patient was actually treated on two key aspects: the best drug to treat his cognitive impairment (donepezil) and key drugs to avoid (amyloid-targeted antibodies). We also compared the clinical trials recommended by OE versus the trial that the patient ended up enrolling in. Concordance was defined by an exact or near-exact match to what was actually done clinically. The AI responses were also evaluated by a subspecialist from an outside institution who was not involved in the patient’s care for depth and logic. The role of the external specialist was to evaluate what had been missed by OE and which recommendations could be improved. He had access to both the prompt (which contained case histories) and OE's summary responses, but did not have access to the patient's medical record. No standardized evaluation framework was used since, as stated above, we looked for an exact or near-exact match with the standard of care.

## Results

We first input a de-identified case summary into OE as shown below. We kept it short but with relevant details to mimic what a non-specialist clinician might do at a point-of-care setting to get a quick response. The questions asked of OE are termed prompts and are in table format. The responses provided by OE are given within quotations, and phrases bolded by OE’s response are bolded here also. Below OE's responses, we provide the degree of concordance between OE's recommendations and what was actually done clinically (Table 1).

**Table 1:** Initial case summary prompt given to OpenEvidence with the patient's symptoms and history

A 75-year-old male with a history of supraventricular tachycardia, sleep apnea, hyperlipidemia, prediabetes, depression, and insomnia presents with a two-year history of memory concerns. He has no other neurological symptoms. He is on atorvastatin, mirtazapine, lithium orotate, citicoline, creatine, and tamsulosin. An MRI done six months earlier revealed mild generalized atrophy and scattered foci of old microhemorrhages intracranially. He has a strong family history of Alzheimer’s and is homozygous for APOE4. What should be the next step in his diagnostic evaluation?

OE's response:

The **next step** in this patient’s diagnostic evaluation should be **formal neuropsychological testing** to objectively characterize the nature and severity of cognitive impairment, followed by **laboratory workup** to exclude reversible causes and **medication review** to identify potentially contributing agents.

**Formal cognitive assessment** is essential given the 2-year history of memory concerns. While brief screening tools like the Mini-Cog or Memory Impairment Screen can detect cognitive impairment, more comprehensive testing with the **Montreal Cognitive Assessment (MoCA)** or **Saint Louis University Mental Status (SLUMS)** examination provides better characterization of cognitive domains and is more sensitive for detecting mild cognitive impairment (MCI). The MoCA has sensitivity of 83.8% and specificity of 70.8% for MCI, while SLUMS is freely available and performs well across diverse populations.

**Medication review** is particularly important in this patient. **Mirtazapine**, while generally having fewer anticholinergic effects than tricyclic and antidepressants, can still contribute to cognitive impairment in elderly patients. Additionally, **lithium orotate** (though not FDA-approved and less studied than lithium carbonate) and the combination of multiple supplements warrant careful evaluation. Anticholinergic and sedative medications are consistently associated with worse attention, processing speed, and executive function in older adults.

**Laboratory evaluation** should include a complete blood count, comprehensive metabolic panel, thyroid function tests, vitamin B12 and folate levels to exclude reversible causes of cognitive impairment. Given his depression history, screening with the Geriatric Depression Scale is appropriate, as depression can mimic or exacerbate cognitive symptoms (sensitivity 86%, specificity 79%).

By and large, OE’s responses matched what was actually done with the patient [7]. We next asked OE about the microhemorrhages seen on the patient's prior MRI scan (Table 2).

**Table 2:** Follow-up prompt given to OpenEvidence on the significance of microhemorrhages

How does the presence of microhemorrhages influence recommendations for further imaging or biomarker studies?

OE’s response:

The presence of cerebral microbleeds (CMBs) **significantly influences both the decision to pursue biomarker testing and eligibility for anti-amyloid therapies,** with the **number, location, and pattern of microbleeds** being critical factors in risk stratification.

Impact on Biomarker Testing Decisions

The presence and pattern of microbleeds can help guide whether amyloid PET or CSF biomarker testing is appropriate. **Strictly lobar microbleeds **(particularly intracortical/juxtacortical) are strongly associated with cerebral amyloid angiopathy (CAA) and amyloid burden, with isolated lobar CMBs or superficial siderosis conferring over 4-fold higher odds of elevated amyloid compared to those without CMBs. In contrast, deep or mixed patterns of microbleeds suggest hypertensive arteriopathy rather than amyloid-related pathology.

In your patient with **scattered foci of old microhemorrhages**, determining the **exact number and anatomical distribution** (lobar vs. deep, intracortical vs. subcortical) is essential before proceeding with biomarker testing. If the microbleeds are predominantly lobar and intracortical, this would support the likelihood of underlying CAA and amyloid pathology, making biomarker confirmations more clinically relevant. However, if they are mixed or predominantly deep, this suggests vascular pathology and would lower the pre-test probability of Alzheimer’s disease despite his APOE4 homozygosity.

Clinically, the patient underwent cognitive testing, cerebral spinal fluid (CSF) biomarker testing, and a brain MRI. Although it was not specified by OE, the patient underwent an MRI scan with susceptibility-weighted imaging (SWI) to fully evaluate the distribution of microhemorrhages. We then provided OE with summary results of key tests (cognitive, CSF, MRI) that had actually been done (Table 3).

**Table 3:** Prompt given to OpenEvidence with test results asking for a differential diagnosis

His MoCA scores were 26 with impaired delayed recall. His CSF p-Tau/Abeta42 ratio was abnormally elevated at 0.098. His brain MR scan showed age-appropriate atrophy, and approximately one hundred cortical and subcortical microhemorrhages in a lobar distribution. There were no additional superficial siderosis or infratentorial microhemorrhages. What is the most likely diagnosis? What is the differential?

OE's response:

Based on this patient’s presentation, the** most likely diagnosis is Alzheimer’s disease with concomitant cerebral amyloid angiopathy (CAA)**, representing a **mixed neurodegenerative-vascular pathology**. The differential includes CAA-related cognitive impairment as a primary driver, vascular cognitive impairment from extensive lobar microbleeds, and the important cannot-miss diagnosis of CAA-related inflammation.

Most Likely Diagnosis: Alzheimer’s Disease with Cerebral Amyloid Angiopathy

Supporting evidence for CAA:

· **Approximately 100 lobar microbleeds** represents severe CAA burden. This extensive lobar-predominant pattern is pathognomonic for CAA.

· **APOE4 homozygosity** is also strongly associated with CAA and lobar microbleeds.

· **CSF profile shows features of both AD and CAA:** The elevated p-tau/Aβ42 ratio indicates AD, but patients with CAA typically show decreased Aβ40 levels compared to AD alone, which helps differentiate the two conditions.

Why this represents mixed pathology:

The coexistence of AD biomarkers (elevated p-tau/Aβ42 ratio) with severe lobar CAA (100 microbleeds) indicates that both parenchymal amyloid plaques (AD) and vascular amyloid deposition (CAA) are contributing to his cognitive impairment. Studies show that 50-85% of CAA patients have decreased Aβ42 with elevated tau markers, suggesting concurrent AD pathology. In APOE4 homozygotes with amyloid pathology, the prevalence of cerebral microbleeds reaches approximately 52% by age 90.

OE's response on the differential diagnosis for our case:

Differential Diagnoses

Other important considerations:

1. **CAA-related cognitive impairment (primary):** While CAA alone can cause cognitive decline through microvascular injury, the elevated p-tau/Aβ42 ratio argues against pure CAA and supports concurrent AD pathology.

2. **Vascular cognitive impairment:** The 100 lobar microbleeds represent severe small vessel disease that independently contributes to cognitive decline. Lobar and mixed microbleed patterns are strongly associated with incident dementia. However, the AD biomarker profile indicates this is not purely vascular.

3. **Medication-related cognitive impairment: Mirtazapine** can contribute to cognitive impairment in elderly patients through sedative and anticholinergic effects. **Lithium orotate** (though not FDA-approved) may also affect cognition. However, these would not explain the AD biomarker profile.

4. **Sleep apnea-related cognitive impairment:** Untreated OSA is associated with increased AD biomarkers (Aβ and tau) and cognitive impairment, particularly in attention and executive function. Sleep fragmentation can impair glymphatic clearance of Aβ and tau. However, this would be contributory rather than the primary diagnosis.

5. **Depression-related cognitive impairment (pseudodementia):** His history of depression on mirtazapine could contribute, but the objective AD biomarkers and extensive structural pathology make this unlikely as the primary diagnosis.

Cannot-Miss Diagnosis:

6. **CAA-related inflammation (CAA-ri):** This is critical to exclude given his presentation. CAA-ri typically presents with:

 a. Subacute cognitive decline or behavioral changes (present in this patient)

 b. Very high lobar microbleed burden (median 207, range 33-811) - **this patient has ~100**

 c. **ARIA-E features:** parenchymal white matter hyperintensities (89%), sulcal hyperintensities (78%), gyral swelling (86%)

 d. Leptomeningeal enhancement (70% sensitivity)

 e. APOE4 homozygosity (34% of CAA-ri patients)

**Key distinguishing features:** The MRI report states ‘age-appropriate atrophy’ without mention of asymmetric white matter hyperintensities, gyral swelling, sulcal hyperintensities, or leptomeningeal enhancement - features that would be expected in CAA-ri. However, **a careful re-review of the MRI specifically looking for ARIA-E features is warranted** given the extensive microbleed burden and APOE4 homozygosity.

Since AI LLM’s answers are prompt-dependent, we probed it further with regard to the patient’s exact diagnosis (Table 4).

**Table 4:** Prompt to OpenEvidence asking to refine the diagnosis

Does he have Possible or Probably CAA? Does he have MCI or Alzheimer’s dementia?

OE's response:

**CAA Diagnosis: Probable CAA** by Boston Criteria version 2.0

**Cognitive Diagnosis: Mild Cognitive Impairment (MCI) due to Alzheimer’s disease**, not dementia

The actual diagnosis given by the treating physician (probable CAA and Mild Cognitive Impairment due to Alzheimer’s disease) matched the diagnosis provided by OE. The differential diagnoses also matched the conditions considered by the treating clinician.

We next prompted OE to provide us with a care plan for the subject (Table 5).

**Table 5:** OpenEvidence prompt asking for the next steps in the care plan

What are the next steps in his care plan?

OE's response:

Based on this patient’s diagnosis of **MCI due to Alzheimer’s disease with probable CAA,** the care plan focuses on **vascular risk factor management, lifestyle interventions, medication optimization, and close monitoring,** as anti-amyloid therapy is contraindicated due to his extensive microbleed burden.

Recommendation for this patient:

**Given his biomarker-confirmed AD, APOE4 homozygosity** (which may predict better response), and **relatively preserved cognition** (MoCA 26), a trial of **donepezil 5-10 mg daily** is reasonable after thorough discussion of limited evidence, off-label use, and potential side effects. Monitor for GI side effects and cardiac conduction abnormalities (baseline ECG recommended).

This recommendation also matched quite closely with what was actually done for the subject in clinical practice. His actual care plan was as follows: He was started on donepezil 5 mg for 6 weeks, which was then increased to 10 mg daily. His physician also recommended that he avoid medications that increase the bleeding risk. He was advised to control his blood pressure and low-density lipoprotein. He was instructed to exercise 30 to 45 minutes daily, engage in intellectual stimulation, social activities, and follow a Mediterranean diet. He was counselled against the use of amyloid-targeted therapies (ATTs).

We then prompted OE to provide a prognosis for the patient (Table 6).

**Table 6:** OpenEvidence prompt asking about his prognosis

What is his prognosis?

As a response, OE provided a table, which is shown below (Table 7).

**Table 7:** Summary prognosis given by OpenEvidence Table generated by OpenEvidence to show the patient’s possible outcomes, the estimated risk of them, and key factors that point to that prognosis. MCI: mild cognitive impairment; CAA: cerebral amyloid angiopathy; ICH: intracranial hemorrhages

Outcome	Estimated Risk	Key Factors
Progression to dementia	~73% at 5 years	MCI status, APOE4 homozygosity, CAA burden
Symptomatic ICH	~5-7% per year	~100 lobar microbleeds, APOE4, no cSS (protective)
Accelerated memory decline	High	APOE4 homozygosity, AD+CAA synergy
Mortality (vs. general population)	HR 4.9	CAA diagnosis, microbleed burden
Life expectancy reduction	~5-10 years	MCI/dementia stage, comorbidities

We next asked OE if there were any clinical trials it would recommend for the patient (Table 8).

**Table 8:** OpenEvidence prompt asking for suitable clinical trials

Are there any clinical trials he may be eligible for?

OE's response:

The search returned **180 available trials** across Alzheimer’s disease, MCI, and cerebral amyloid angiopathy. Given this patient’s unique profile-**APOE4 homozygosity, extensive microbleeds (~100), and ineligibility for anti-amyloid therapy**-the most relevant trials from the search results above fall into several categories:

CAA-Specific Trial:

· **NCT05709314** (AMDX-2011P for CAA) - A Phase 2 trial specifically targeting cerebral amyloid angiopathy, which directly addresses his CAA pathology. This may be particularly relevant given his extensive microbleed burden.

We could not ask OE about the availability of trials specific to the patient's zip code due to privacy issues, and hence, its answers were less specific. In actual practice, the patient was referred to the ALN-APP (an investigational RNA interference agent aimed at lowering amyloid-beta) clinical trial, for patients with CAA (NCT06393712) [8]. He was screened but subsequently disqualified due to his inability to lie in a specific position for the study MRI. He was referred to a study examining the efficacy of computerized cognitive training (NCT06601933) [9], which he is currently enrolled in.

## Discussion

In a very short time frame of about three years since launch, OE is being used as a knowledge aid by almost half of all practicing physicians in the US [1-4,10]. This explosive growth clearly highlights the strong unmet need for a rapid point-of-care knowledge co-pilot. However, it also highlights the need to ensure its recommendations are accurate, especially for nuanced or complex questions.

To date, a dozen or so published studies have compared OE versus standard of care utility [2-4,11-12]. As stated previously, a Mayo Clinic study of five cases found OE’s performance to be accurate and evidence-based [3]. A study of the reliability and clinical applicability of three general LLMs versus OE in providing recommendations for knee meniscal pathology (against the gold standard of American Academy of Orthopedic Surgeons Clinical Practice Guidelines) found that OE achieved perfect concordance (9/9, 100%), followed by ChatGPT (8/9, 89%; OpenAI, San Francisco, California, US), Gemini (Google, Mountain View, California, US), and Claude (Anthropic, San Francisco, California, US); both the latter were 7/9, 78% [11]. This study concluded that the outputs of general-purpose chatbots are suboptimal versus OE, which is designed to provide higher-quality evidence-based information. Another recent study found that OE showed consistent, guideline-concordant reasoning across 12 simulated emergency and primary-care otolaryngology cases and that LLMs had higher referral accuracy than a comparison group of emergency room and family medicine physicians [12]. OE can also provide information on clinical trials relevant to the individual and can even locate trials in zip codes close to where the patient lives [4]. As OE becomes fully integrated into electronic medical records, it will make it easier to import full case details to get more precise responses. 

To our knowledge, this is the first case example of OE’s utility in a patient with two common comorbid neurological conditions. Moderate-to-severe CAA pathology is detected in close to one-quarter of all post-mortem brains and close to half of the brains with Alzheimer’s disease, representing a highly prevalent age-related condition [13]. When present at this severity, it may add to cognitive morbidity and pose a dilemma for the use of ATTs [5,7]. Thus, our case represents two conditions that are commonly encountered in both general practice and in specialist clinics.

At a high level, OE’s diagnosis and much of its care plan were a close match for what was actually done in the clinic [7] by the patient’s provider (a specialist). OE’s response speed, clarity, reasoning, and explanation would likely have been very helpful to a non-specialist or trainee. Although we could not include all the references that OE cited due to lack of space, OE does cite high-quality references and practice guidelines with the level of evidence supporting its key recommendations. OE also provides tables and graphs that offer relevant and rapid knowledge both for care and for general education purposes. Each prompt was written to try to mimic what a clinician would need at the point of care.

However, a review of the case vignette by a co-author (SMG), who is a specialist in stroke and CAA, and is not involved with the patient’s care, identified some areas where OE’s response was overstated, erroneous, or lacked depth. In presenting the expert concerns listed below, we note that OE’s responses are highly dependent on the prompt and that a specialist who asked more detailed prompts would therefore be expected to get better responses than one asking a more generic prompt. OE rarely questioned the completeness of the prompt and usually provided an answer. Here are some examples of points raised.

Neurocognitive evaluation: An important omission was assessment of daily functional skills or instrumental activities of daily living (IADLS), which is necessary for diagnosing MCI vs dementia. There was also no mention of behavioral assessments in areas such as depression or sleep. While the recommendation to obtain neuropsychological testing is accurate, the tools suggested by OE as comprehensive (the Montreal Cognitive Assessment (MOCA) [14] and Saint Louis University Mental Status Examination (SLUMS) [15]) are, in reality, high-level screening tools aimed towards a general practice audience rather than comprehensive diagnostic tools. 

MRI:* *OE’s recommendation for evaluating microhemorrhages did not consider or specify the type of T2*-weighted MR sequence used (e.g., SWI vs conventional gradient echo (GRE)), which has large effects on sensitivity [16]. This would have needed a second prompt asking what type of MRI is most sensitive for such pathology.

CSF:* *OE took the CSF results we uploaded as verbatim without questioning what assay method was used and what were the results of the individual analytes used to compute the ratio. OE also interpreted the elevated CSF p-tau/Ab42 finding as diagnostic of Alzheimer’s disease pathology (which, to be fair, is also commonly done by many Alzheimer’s disease-centric doctors), but CAA alone can result in elevated ratios. Again, this higher level of reasoning would have required multiple prompts.

Diagnosis and differential diagnosis: The initial diagnosis given by OE of Alzheimer’s disease was changed to MCI upon further probing without providing a specific basis (e.g., was a cutoff on MoCA or functional skills used by OE to decide if the subject was MCI or dementia). Traditionally, a key boundary between the terms MCI and Alzheimer’s disease dementia was whether or not a subject had preserved independence or not. However, the newer revised Alzheimer’s Association criteria use the term Alzheimer’s disease more broadly, even for earlier stages, and emphasize biomarker positivity. It would have been useful to know how much OE’s AI relied on these newer biomarker-based diagnostic terminology versus the older National Institute of Neurological Disorders and Stroke (NINDS) or Diagnostic and Statistical Manual of Mental Disorders, Fifth Edition, Text Revision (DSM-5-TR) psychiatric criteria [17,18], and why it did so. Further, CAA diagnostic criteria specify *strictly* lobar microbleeds, not *predominantly* lobar. In the discussion of CAA-ri, “Subacute cognitive decline or behavioral change (present in this patient)” is an incorrect statement. The vignette reported a two-year course of cognitive decline, which isn’t subacute. These appeared to represent knowledge gaps and a lack of specificity.

Medications: OE’s caution that mirtazapine may impair cognition, while drawn from the warnings on the package insert, is superficial, and does not reflect the geriatric risk-benefit trade-offs (sedation/falls versus improved sleep/mood) done in practice. A deeper logical analysis of the pros and cons of all the subjects' medications would have required additional prompts to evaluate.

Management: Multiple responses by OE refer to preventing “recurrent stroke,” “ICH recurrence," or “secondary prevention,” but these responses are misleading, as the patient has not had an initial clinical stroke or macrohemorrhage. This leads to some misstatements, e.g., “target LDL <70 mg/dL for secondary prevention”. OE’s caution that anticoagulation is contraindicated is an overstatement, as current practice is to weigh the risks against the benefits of anticoagulation [19]. There is also no consensus that annual MRIs in CAA are required indefinitely, though in a patient with 100+ microhemorrhages, frequent follow-up may be prudent. The listed quantitative MRI markers and fluid biomarkers are not all usually done in routine practice, though this patient did undergo both, as he was evaluated at a specialty clinic.

Prognosis: The annual risk of symptomatic ICH for CAA patients with no prior ICH or cortical superficial siderosis (sometimes referred to as “microbleed-only” CAA) appears to be approximately 1.2%, not 5% [12]. AI-generated numerical prognoses may be suboptimally calibrated and must be independently verified.

Limitations

This case presents several limitations. This is a single retrospective case example. It did not include a blinded evaluation using a structured rating scale but instead relied on a direct qualitative concordance with what was actually done in practice. We used deidentified generic prompts rather than inputting the actual full case from the electronic record for privacy reasons. LLMs are prompt-dependent, so it’s possible that a more personalized role-play prompt (“assume you are the best cognitive expert in the country, how will your management plan change if you are referred this patient?”) could have generated an expert-level management plan and enhanced OE’s accuracy even more. Physicians should consider such prompt role play. No generalized claims of accuracy or safety can be made from a single case, other than that it is idea-generating and supportive of prior published studies. A structured evaluation in larger prospective blinded studies across diverse settings is needed. Last but not least, it must be borne in mind that tools like OE are meant as knowledge copilots (educational aids), not as autonomous agents, and that all such information should be verified by a clinician.

## Conclusions

Overall, with the AI responses, more information than was originally considered was present. While there were some suboptimal answers, OE used as a knowledge aid (instead of as "the best answer") can help inform physician decisions by giving them more information than they may have considered. Our case adds to the prior literature supporting OE’s utility as a knowledge aid for the generalist. With its vast, rapidly growing search database, OE is well-suited to create knowledge graphs that can identify gaps in our evidence base and annotate those gaps with expert input. Further studies evaluating how OE may enhance and support education and care will be useful.
